# Probing Carbon Utilization of *Cordyceps militaris* by Sugar Transportome and Protein Structural Analysis

**DOI:** 10.3390/cells9020401

**Published:** 2020-02-10

**Authors:** Kanokwadee Sirithep, Fei Xiao, Nachon Raethong, Yuhan Zhang, Kobkul Laoteng, Guang Hu, Wanwipa Vongsangnak

**Affiliations:** 1 Center for Systems Biology, School of Biology and Basic Medical Sciences, Soochow University, Suzhou 215123, China; kanokwadee.s@ku.th (K.S.); xiaofei@suda.edu.cn (F.X.);; 2 Genetic Engineering and Bioinformatics Program, Graduate School, Kasetsart University, Bangkok 10900, Thailand; 3 Department of Zoology, Faculty of Science, Kasetsart University, Bangkok 10900, Thailand; nachonase@hotmail.com; 4 Functional Ingredients and Food Innovation Research Group, National Center for Genetic Engineering and Biotechnology (BIOTEC), National Science and Technology Development Agency (NSTDA), Pathum Thani 12120, Thailand; 5 Omics Center for Agriculture, Bioresources, Food, and Health, Faculty of Science, Kasetsart University (OmiKU), Bangkok 10900, Thailand

**Keywords:** *cordyceps militaris*, carbon metabolism, comparative genomics, protein structure, sugar transporter, network analysis

## Abstract

Beyond comparative genomics, we identified 85 sugar transporter genes in *Cordyceps militaris*, clustering into nine subfamilies as sequence- and phylogenetic-based functional classification, presuming the versatile capability of the fungal growths on a range of sugars. Further analysis of the global gene expression patterns of *C. militaris* showed 123 genes were significantly expressed across the sucrose, glucose, and xylose cultures. The sugar transporters specific for pentose were then identified by gene-set enrichment analysis. Of them, the putative pentose transporter, CCM_06358 gene, was highest expressed in the xylose culture, and its functional role in xylose transport was discovered by the analysis of conserved structural motifs. In addition, a battery of molecular modeling methods, including homology modeling, transport pathway analysis, residue interaction network combined with molecular mechanics Poisson–Boltzmann surface area simulation (MM-PBSA), was implemented for probing the structure and function of the selected pentose transporter (CCM_06358) as a representative of sugar transportome in *C. militaris*. Considering the network bottlenecks and structural organizations, we further identified key amino acids (Phe38 and Trp441) and their interactions with other residues, contributing the xylose transport function, as verified by binding free energy calculation. The strategy used herein generated remarkably valuable biological information, which is applicable for the study of sugar transportome and the structure engineering of targeted transporter proteins that might link to the production of bioactive compounds derived from xylose metabolism, such as cordycepin.

## 1. Introduction

*Cordyceps militaris* is an obligate pathogen of insects that has a long history of use for herbal drugs in Asia [1]. This pathogenic fungus and related strains are being exploited as natural sources for production of bioactive compounds [2]. Of them, cordycepin is an important bioactive compound found in *C. militaris*, which has a broad range of biological activities against various types of human cancer, such as ovarian [3] and lung [4]. Moreover, a novel anti-adipogenic potential of cordycepin was discovered, which induces metabolic reprogramming and browning in white adipose tissues through the activation of AMP-activated protein kinase-dependent pathway [5,6]. These medicinal significances have expanded innovative implications of *C. militaris* for pharmaceutical and functional food industries [7]. Increasing demands have led to the cultivation development for production of high quality of *C. militaris* cell-mass rich in cordycepin. Not only for the parasitic growth in lepidopteran larva, the artificial cultivation is of particular interest in the mass production at large scale [1]. Much attention has been paid to the investigation in cordycepin biosynthesis using different feedstocks or different cultivation modes. Apart from the common use of rice grains for the cultivation of *C. militaris* by solid-state fermentation (SSF), sugars with different carbon atoms were also investigated for the liquid cultivation with static mode [8]. Intriguingly, glucose and sucrose were the best carbon sources, which potentially triggered the downstream carbon metabolism for cell growth and cordycepin overproduction in *C. militaris* [9]. However, the production of cordycepin on xylose seems to be promising, even though it was a poor carbon source for fungal growth.

Renewable feedstocks, including agricultural and agro-industrial by-products, are of current interest in the production of diversified bioproducts [10]. The bioconversion of the complex carbohydrate polymers to monomer and dimer sugars, such as glucose and sucrose, respectively, has been thought to be an economically feasible process for industrial practice [11]. In addition, the cellulosic sugars have been documented as renewable options for the production of medicinal biopolymers, such as beta-glucan and exopolysaccharides by submerged cultivations of *C. militaris* and *Cordyceps sinensis* [12]. This finding indicates a prospect in the sustainable production of the high-value metabolites by *Cordyceps* spp. using renewable feedstocks. Accordingly, the major metabolic pathways underlying sugar metabolism in *C. militaris* have been intensively studied through transcriptome analysis [9]. The study uncovered a hint of the global metabolic response to specific carbon sources during cordycepin overproduction stage.

Apart from the metabolic reactions relevant to sugar metabolism, how the sugar transports across the cellular membrane or compartment remains unexplored in *C. militaris*. In filamentous fungi, the sugar transporters were mostly studied in Ascomycetes, such as Aspergilli, and thus the term “sugar transportome” has been raised to represent whole sugar transporters, which was investigated through advanced bioinformatics and next generation sequencing technology [13]. As a result, a number of sugar transporters (86 genes) were identified in the *Aspergillus niger* genome. Of which, 16 transporters were specific for monosaccharides, such as glucose, fructose, galacturonic acid and xylose, whereas a smaller number of transporters (10 genes) responsible for disaccharide sugars (sucrose and maltose) was detected [13]. In addition, the sugar transporters in some industrial strains, such as *Saccharomyces cerevisiae* and *Trichoderma reesei*, have been reported [14,15]. Only a few of the sugar transporters have been described in entomopathogenic fungi, such as trehalose transporter in *Beauveria bassiana* BbAGT1 and raffinose transporter in *Metarhizium robertsii* Mrt [16,17]. This study was therefore devoted to investigating the sugar transporter function by integrative analysis of sugar transportome, global transcriptome, and protein structure with the overall aim of probing carbon utilization in *C. militaris*. Based on the significant differential expression data of the *C. militaris* cultivation on xylose when compared to those using sucrose and glucose, the functional and structural annotations of the targeted transporter was implemented. In addition, the study of transport pathway, residue interaction network, and binding free energy analysis of the targeted transporter elaborated the way for structure-based modeling by predicting important residues and interactions, for now providing mechanistic understanding of the sugar transporter function.

## 2. Materials and Methods

### 2.1. Construction of Sugar Transportome

**Figure 1:**
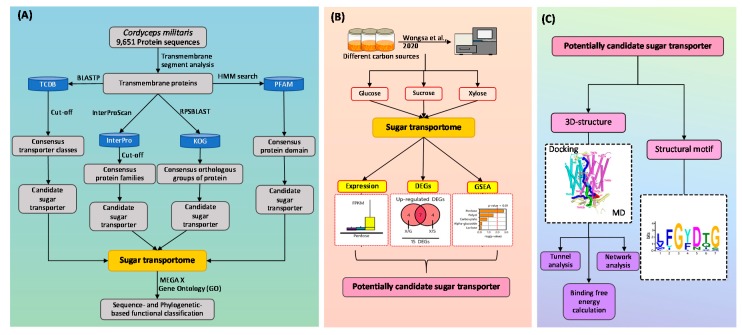
Systematic workflow of integrative analysis of sugar transportome, global transcriptome and protein structure in *Cordyceps militaris*. Note: (**A**) The construction of sugar transportome by transmembrane segment analysis and consensus features identification against *C. militaris* genome [18]; (**B**) integrative analysis of sugar transportome and global transcriptome data through differentially expressed genes (DEGs) identification and gene set enrichment analysis (GSEA) across different carbon sources for identifying a candidate sugar transporter; (**C**) the three-dimensional (3D) structural analysis and structural motif analysis of the targeted transporter. The 3D-structural analysis includes modeling, docking and molecular dynamics (MD) simulation, followed by tunnel, network, and binding free energy analysis.

Initially, 9651 protein sequences from *C. militaris* genome [18] were applied to transmembrane segment analysis to obtain transmembrane proteins with default parameters [19]. Then, the obtained transmembrane proteins were subjected to functional annotation by the four different protein databases for further sugar transporter identification as shown in Figure 1. Firstly, we searched the consensus sugar transporter domain in Protein domain (PFAM) database (PF00083) against transmembrane proteins [20]. Secondly, we identified candidate sugar transporters from transmembrane proteins based on consensus protein families of sugar transporters in Protein families in InterPro database (IPR003663, IPR004853, IPR005828, IPR005829, IPR007271, and IPR011701) using InterProScan with an E-value of 1E-05 as a cut-off [21]. Then, Reverse PSI-BLAST (RPSBLAST) was applied to obtain transmembrane proteins for identifying candidate sugar transporters based on eight eukaryotic orthologous groups of proteins from Eukaryotic orthologous groups (KOG) database (KOG0254, KOG1444, KOG1441, KOG2234, KOG0252, KOG0769, KOG4332, and KOG056) [22]. Finally, candidate sugar transporters were also identified by querying protein sequences from *C. militaris* genome against protein sequences of transporters existed in transporter classification database (TCDB) [23] using BLASTP with E-value of 1E-05 and identity of 25% as cut-off values [24]. Particular putative transporters, which functionally classified in classes i.e., 2.A.1, 2.A.2, 2.A.7, 2.A.16, 2.A.29, 2.A.50, and 2.A.96, were manually selected as candidate sugar transporters. Overall identified candidate sugar transporters were overlapped to get non-redundant sugar transporters, which were then included in the sugar transportome of *C. militaris*.

### 2.2. Multiple Sequence Alignment and Structural Motif Analysis

Multiple sequence alignment was applied to non-redundant sugar transporters for sequence- and phylogenetic-based functional classification using MEGA X [25]. Each non-redundant sugar transporter was then grouped into putative subfamilies based on the phylogenetic relationship and gene ontology (GO) [26]. Besides the functional classification, the multiple expectation maximization for motif elicitation (MEME) suite was applied for structural motif analysis of protein sequences of selected sugar transporters and known/putative sugar transporter sequences as listed in Table S1 [27].

### 2.3. Gene Set Enrichment Analysis

To explore the transcriptional response of sugar transportome, the transcriptome data obtained from *C. militaris* cultures using different carbon sources by Wongsa et al. (2020) [8] were mapped against non-redundant sugar transporters. Then, the differentially expressed genes (DEGs) were defined by the altered expression level with a |log2 fold change| ≥ 2 and a false discovery rate (FDR) ≤ 0.001. The identified DEGs were further investigated through consensus gene set enrichment analysis for identification the transcriptional response of sugar transportome in different carbon sources [28]. This was performed by statistical analysis (*p*-value) of transcript levels of DEGs, using a set of sugar transporters or their subfamilies as the inputs. The sugar transporters or subfamilies having distinct-directional *p*-values below 0.01 were then identified as significant sugar transporters or subfamilies [28,29]. Subsequently, a significant sugar transporter with the highest expression level as indicated by the value of fragments per kilobase of transcript per million mapped reads (FPKM) was selected as a potentially candidate sugar transporter for further protein structural analysis.

### 2.4. Structural Modeling and Molecular Docking

The protein sequence of a potentially candidate sugar transporter was retrieved from the UniProtKB database [30]. A similarity search for CbDPEase was performed by the MODDELLER version 9.17 [31] to find potentially related sequences of known structures as suitable templates. Thus, the X-ray structure of STP10 (PDB ID: 6H7D) was chosen as a template for modeling a potentially candidate sugar transporter. The disordered loop region of N-terminus was reconstructed in the modelled structure of a potentially candidate sugar transporter by applying spatial restraints and energy minimization using MODELLER. Subsequently, the targeted sequence and template were aligned using MODELLER, and a three-dimensional (3D) model was constructed. The constructed model was energy minimized in a GROningen MAchine for Chemical Simulations (GROMACS) force field using steepest descent minimization algorithms. After geometry optimization, most structural features could be observed in the model.

To study the interaction and binding mode of sugar with the potentially candidate sugar transporter, molecular docking was carried out using AutoDock 4.2 [32]. The 3D-structure of the potentially candidate sugar transporter was depicted from the homology modeling, and the sugar molecule structure was obtained from PubChem [33]. All water atoms were removed, and hydrogen atoms were added into the structures using the PyMOL (version 0.99; DeLano Scientific, San Carlos, CA, USA). The non-bonded interactions were calculated using all atomic parameters of AutoDock based on the AMBER force field, since they reproduced the best crystal ligand conformations. Implicit solvation parameters were added from the AutoDock Tools package. Ligand non-polar hydrogen atoms were marked as atom type ‘X’. Docking grids for carbon, oxygen, polar, and non-polar hydrogen atoms were calculated by AutoGrid, which was also a part of AutoDock. Grid maps contained points for the potentially candidate sugar transporter and the compound to constrain them within the docking cavity. Two candidate docking cavities were chosen as the opening and bottleneck of the tunnel. The root mean square deviation (RMSD) and affinity energy were used in selecting the best interaction poses.

### 2.5. Bilayer Construction and Molecular Dynamics Simulation

CHARMM (CHARMM36 force field [34]) simulations were set up using the Chemistry at HARvard Macromolecular Mechanics-Graphical User Interface (CHARMM-GUI) [35] together with the membrane builder [36]. Pre-assembled POPE bilayers containing 100 lipids per leaflet were generated via the CHARMM-GUI. The bilayers were immersed in water such that there was approximately a 15 Å layer of water on each side. The minimization and equilibration were performed using the standard GROMACS inputs of the CHARMM-GUI. The simulations were then run using the GROMACS.

Additionally, the constructed model of the potentially candidate sugar transporter docking with sugar molecule was further embedded into a POPE lipid membrane, and solvated in water with K^+^ ions concentration of 1.2 M (as calculated by the number of ions relative to the number of water molecules in the system) and Cl^−^ ions to neutralize the system. Later, molecular dynamics (MD) simulation was further carried out for 100 ns using the GROMACS software package (version 5.1) [37] using a timestep of 2 fs. The non-bonded interaction potential was smoothly switched off between 10 and 12 Å, beyond which Coulombic interactions were treated with the particle-mesh Ewald method [38]. A temperature of 303.15 K was maintained via the Nosé-Hoover thermostat [39]. The simulation system was coupled semi-isotropically with the Parrinello−Rahman barostat to maintain a pressure of 1 atm independently in the bilayer normal and lateral directions [40]. The atomistic CHARMM36 force field was used to model POPE and transferable intermolecular potential three-point model (TIP3P) was used to model water [41].

### 2.6. Transport Pathway Identification and Residue Interaction Network Analysis

CAVER 3.0 [42] was used for identifying the transport pathways, called tunnels, based on the modeling structure of the potentially candidate sugar transporter. The probe radius and the clustering threshold were set to 0.8 and 4.5 Å, correspondently. The shell depth and radius were set to 4 and 3 Å, respectively, and other parameters were used as default settings throughout the calculation.

The residue interaction network (RIN) of the potentially candidate sugar transporter was constructed from its protein structure by considering individual amino acids as nodes that are connected by edge over the non-covalent interaction between main chain (mc) and side chain (sc), including van der Waals contacts, hydrogen bonds, overlaps of van der Waals radii, and a combination of any of the previous three interactions. Based on the RIN of the potentially candidate sugar transporter, the network matrix of betweenness was calculated to measure the centrality of each residue. The betweenness centrality B*_k_* of a node *k* is the number of times that a node is included in the shortest path between each pair of nodes, normalized by the total number of pairs. It is defined as:

(1) $$B_{k}=\sum_{s\neq n\neq t}\left(\sigma_{st}(k)/\sigma_{st}\right)$$

where *s* and *t* are nodes in the network other than *k*, *σ_st_* denotes the number of shortest paths from *s* to *t*, and *σ_st_* (*n*) is the number of shortest paths from *s* to *t* that *k* lies on. The betweenness centrality of a node reflects the amount of control that this node exerts over the interactions of other nodes in the network. The construction of RIN for the potentially candidate sugar transporter and the network centrality analysis were performed in Cytoscape using the RINalyzer and structureViz2 apps [43].

### 2.7. Binding Free Energy Calculation

Binding free energy calculation was performed by the molecular mechanics Poisson–Boltzmann surface area (MM-PBSA) method available in GROMACS software packages using g_mmpbsa tool [44]. In this study, g_mmpbsa tool used the GROMACS software version 5.1.4 and APBS (adaptive Poisson–Boltzmann solver) version 1.4.1 [45,46]. The last 50 ns for simulation of complex was chosen as an equilibrium part of the trajectory for energy analysis.

The binding energy (ΔG_bind_) consists of three energetic terms, i.e., potential energy in vacuum, polar-solvation energy (ΔG_polar_), and non-polar solvation energy. The potential energy in vacuum ΔE_MM_ including electrostatic interaction (ΔE_elec_) and van der Waals interaction (ΔE_vdW_). The calculation of non-polar solvation energy was based on SASA model (ΔG_SASA_). The entropy contribution was not included in the calculation of binding energy.

## 3. Results

### 3.1. Characteristics and Consensus Features of Sugar Transportome

Since sugar transporters are a substantial group of transmembrane proteins, therefore 9651 protein sequences of *C. militaris* were initially subjected to transmembrane (TM) segment analysis. After discarded non-transmembrane proteins, 2284 transmembrane proteins (23.67% of *C. militaris* genome) were identified and subjected to functional annotation against the four different protein databases for further sugar transporter identification. By searching the consensus sugar transporter domain (PF00083) against the 2284 transmembrane proteins, only 49 candidate sugar transporters were derived based on PFAM annotation. Further, 203 candidate sugar transporters were additionally identified according to the alternative consensus features of sugar transportome, including six protein families from InterPro, eight eukaryotic orthologous groups of proteins from KOG, and seven transporter classes from TCDB as presented in Table 1 (detailed in Table S2). These candidate sugar transporters were hereby overlapped under statistical significance consequently yielding a total of 85 non-redundant sugar transporters of *C. militaris*, which were included in the sugar transportome. Coincidentally, the gene number in sugar transportome is closed to the previous study in *A. niger*, which 86 putative sugar transporters were identified by in silico analysis [13].

### 3.2. Functional Classification of C. militaris Sugar Transporters

**Figure 2:**
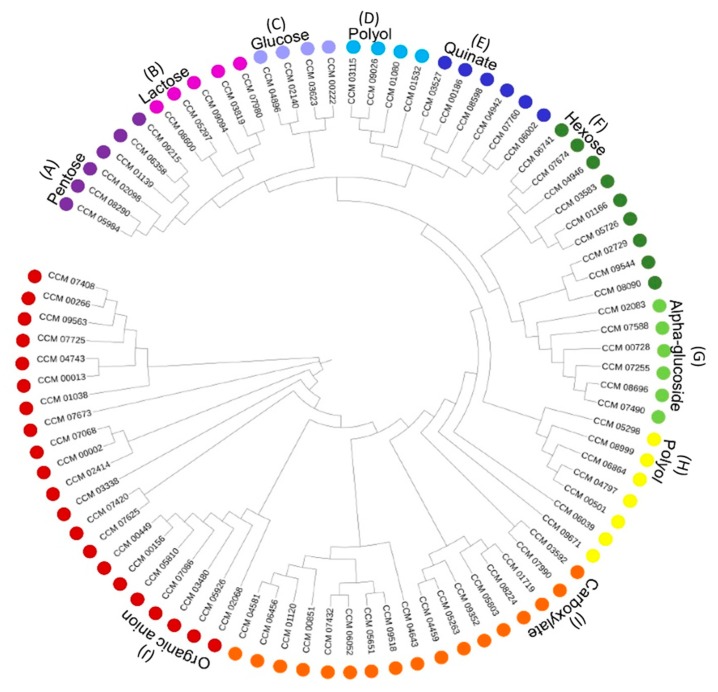
Phylogenetic-based classification of subfamilies of sugar transportome in *C. militaris*. Note: 85 sugar transporters of *C. militaris* were classified into nine subfamilies; (**A**) pentose (six genes); (**B**) lactose (five genes); (**C**) glucose (four genes); (**E**) quinate (six genes); (**F**) other hexose sugars (nine genes); (**G**) alpha-glucoside (six genes); (**D**,**H**) polyol (11 genes); (**I**) carboxylate (17 genes); (**J**) organic anion (21 genes) by sequence- and phylogenetic-based functional classification using MEGA X [25] and gene ontology (GO) [26].

Besides the consensus features, 85 sugar transporters were divided into nine subfamilies by sequence- and phylogenetic-based functional classification. The result is illustrated in Figure 2. This result revealed nine putative subfamilies of *C. militaris* sugar transportome according to gene ontology (GO) of each sugar transporter, such as alpha-glucoside transmembrane transporter activity (GO: 0015151), pentose transmembrane transporter activity (GO: 0015146), and hexose transmembrane transporter activity (GO: 0015149). Interestingly, 19 sugar transporters were classified based on sugar specificity into monosaccharide subfamilies, including pentose (six genes), glucose (four genes), and other hexose sugars (nine genes). In addition, 11 sugar transporters were classified into disaccharide subfamilies, including lactose (five genes) and alpha-glucoside (six genes). Notably, more sugar transporters were presented within the other sugar subfamilies, including polyol (11 genes), quinate (six genes), organic anion (21 genes), and carboxylate (17 genes). This result provides a basis of sugar transporters of *C. militaris* and also supports the versatile capability of the fungal cell in coping with diversified simple sugars for growth as previous studies [8]. However, there is not much to report regarding the polyol transport of the fungal group. Actually, this insect pathogenic fungus has capability in the production of mannitol which is a sugar alcohol with commercial interest in food products and also for medicinal uses [9]. It is noteworthy that further investigation in the functional role of the *C. militaris* genes coding for putative polyol transporters would be valuable for industrial viewpoints.

### 3.3. Transcriptional Response of C. militaris Sugar Transportome in Different Carbon Sources Uncovered Potentially Candidate Sugar Transporter for Xylose Utilization

**Figure 3:**
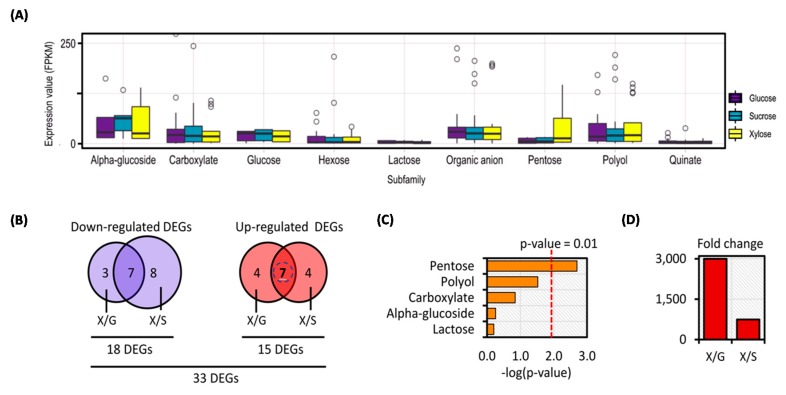
Transcriptional response of *C. militaris* sugar transportome in different carbon sources uncovered CCM_06358 as a potential candidate sugar transporter for xylose utilization. Note: (**A**) Distribution of expression values (FPKM) across different carbon sources and subfamilies; (**B**) number of differentially expressed genes (DEGs) between the xylose culture versus the glucose culture (X/G) and the xylose culture versus the sucrose culture (X/S); (**C**) the top-five enriched subfamilies of the X/G dataset; (**D**) fold changes of CCM_06358 were identified in the X/G and X/S datasets.

According to the diversity of sugar transporters that existed in *C. militaris*, an interesting biological question was raised regarding how individual subfamily of sugar transportome respond to different carbon sources, particularly in the biosynthesis of primary metabolites for cell growth through carbohydrate, lipid, and protein metabolisms as well as for the production of secondary metabolites. Therefore, all 85 sugar transporters were analyzed by integration with the gene expression data of *C. militaris* TBRC7358 [8]. Of which, 79 sugar transporters across 123 genes were significantly expressed among three experimental sets of the sucrose, glucose, and xylose cultures as listed in Table S3. The expression values (FPKM) of these genes were plotted against the nine subfamilies of *C. militaris* sugar transportome as shown in Figure 3A. Among the different carbon sources, the genes of sugar transporters in each subfamily were expressed in a similar fashion except for those involved in the pentose subfamily, which were highly expressed in the xylose cultivation (Figure 3A). These transcriptional changes might participate in the xylose utilization of *C. militaris* for either cell growth or other metabolism.

To explore this finding, the 123 genes among sugar transporters were subjected to the analysis of DEGs by the pairwise comparisons of the xylose culture against the other two carbon sources cultures (i.e., glucose and sucrose). Under the thresholds of |log2 fold change| ≥ 2 with FDR cut-off ≤ 0.001, 33 DEGs were identified, where 15 DEGs were upregulated and 18 DEGs were downregulated when compared the xylose culture with the other cultures using glucose and sucrose (Figure 3B and Table S3). Using the sugar transportome of *C. militaris* as a gene set, all DEGs (33 genes) were further explored through consensus gene set enrichment analysis for identification of specific sugar transporters responsible for xylose utilization [28]. As shown in Figure 3C, it is clearly seen that among the upregulated DEGs involved in the subfamilies of pentose transporters (i.e., CCM_06358, CCM_09215, and CCM_05984) and polyol transporter (i.e., CCM_06864) were significantly enriched in the xylose culture when compared with the other cultures using glucose and sucrose. Very interestingly, one of these pentose transporters, CCM_06358, showed the highest expression in the xylose culture (FPKM of 1170.18). It was significantly upregulated in the xylose culture with the fold changes of 3000.46 and 740.62 when compared to the glucose and sucrose cultures, respectively (Figure 3D). This result suggests that CCM_06358 might be a potential candidate in relation to transport of a particular carbon source (i.e., xylose). In *A. niger*, two pentose transporters, i.e., An11g09600 and An03g01620, were highly expressed in the xylose and arabinose cultures, which were explained by the control of specific transcription factor (TF) as demonstrated by using a set of TF mutants [13]. Possibly, the upregulation in a set of DEGs found in the xylose culture of *C. militaris* indicated the presence of a coregulatory mechanism in controlling the sugar transport towards cellular processes, such as growth, development, stress response, and secondary metabolite production. The xylose not only supports the growth of *C. militaris*, but is also a precursor of the biosynthesis of cordycepin, which is a biologically active compound with industrial interest. It has been recently reported that the cultivation of *C. militaris* on xylose as an alternative carbon source exhibited high production yield of cordycepin on dry biomass. In addition, the upregulated expressions of genes involved in pentose and glucoronate interconversions have been documented by Wongsa et al. (2020) [8]. Accordingly, the relationship between the xylose and cordycepin production was not as straightforward as the precursor role and its derived metabolisms mentioned earlier [8], but it might have a hint of the mechanism controlling at a global level, such as transcriptional and metabolic controls as well as the membrane-associated participation by relevant transporter(s). Gathering the informative data that exists, this might simply draw a linkage of xylose transport and consequently its metabolic behaviors through at least transcriptional regulation. However, the xylose-response mechanism in the fungal cells might be a sophisticated process, and thus the experimental research is additionally required to gain better understanding.

### 3.4. Putative Xylose Transport Function of CCM_06358 Revealed by the Presence of Highly Conserved Structural Motifs

**Figure 4:**
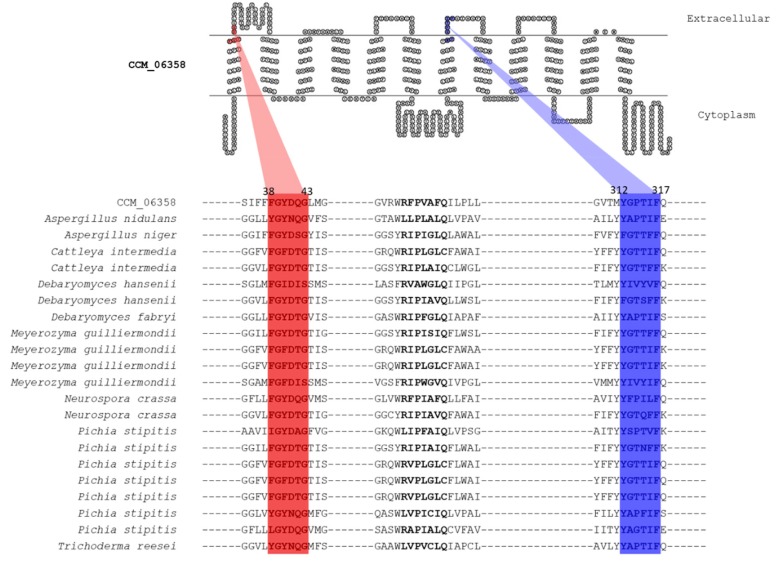
Multiple sequence alignment between CCM_06358 and the selected known/putative sugar transporters shows the highly conserved structural motifs for sugar transport function. Note: The most signatures in two conserved motifs were identified by MEME [27]. These were Phe38-Gly39-Tyr40-Asp41-Gln42-Gly43 (FGYDQG) and Tyr312-Gly313-Pro314-Thr315-Ile316-Phe317 (YGPTIF) in the first and seventh transmembrane (TM) helices, as red- and blue-highlighted letters, respectively. This visualization was created based on hidden Markov model for topology prediction (HMMTOP) transmembrane prediction and display tool (www.sacs.ucsf.edu/cgi-bin/hmmtop.py) [50].

To characterize the functional role of CCM_06358, a multiple sequence alignment between known/putative sugar transporters and CCM_06358 was performed by MEME suite [27]. Strikingly, we found the highly conserved structural motif, Phe38-Gly39-Tyr40-Asp41-Gln42-Gly43 (FGYDQG), at the first transmembrane helices of CCM_06358 under the E-value of 4E-42. The result is illustrated in Figure 4. The identified conserved structural motif was considered as a well-defined specific sequence shared among the sugar transporters in fungi [47]. More specifically, the residue Phe38 has been postulated to display a crucial role as a xylose recognition site in *Candida intermedia* glucose-xylose symporter 1 (GXS1) and *Pichia stipitis* xylose uptake 3 (XUT3). The point mutation at such residue significantly altered the carbon utilization profiles in *C. intermedia* [47,48]. In addition, another highly conserved structural motif, Tyr312-Gly313-Pro314-Thr315-Ile316-Phe317 (YGPTIF), presented at the seventh transmembrane helices of CCM_06358 was discovered under the E-value of 1.4E-39. This motif was also highly conserved in the xylose transporters of lignocellulose-degrading fungi, such as *A. niger* and *T. reesei* [49]. These identified structural motifs shed light on the selective transport function of CCM_06358 for xylose molecules. Nevertheless, a variability in some residues of the conserved motifs was also observed among different fungi (Figure 4), which has possibly arisen from their genetic evolution and might contribute to the substrate specificity and affinity.

### 3.5. Annotated Molecular Structure and Transport Pathway of a Selected Pentose Transporter, CCM_06358

**Figure 5:**
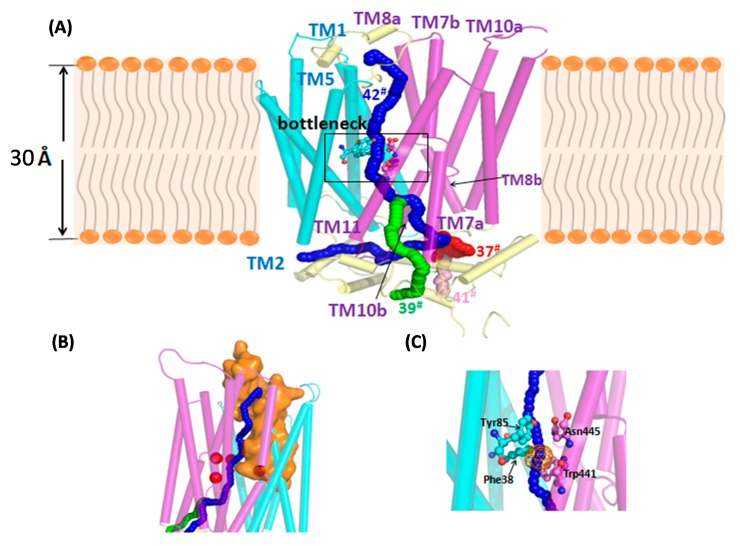
Structural annotation and transport pathway analysis of CCM_06358. Note: (**A**) The modeled structure of CCM_06358 containing 12 transmembrane (TM) helices and 3D-visualization of its four candidate tunnels. Lipid molecules are represented by simple polar heads and hydrophobic tails; (**B**) the tunnels that shared the same entrance are shown in orange; (**C**) the same bottleneck residues are shown in licorice, while the orange wireframe indicates the pocket formed by the four bottleneck residues represented by red spheres i.e., Phe38, Tyr85, Trp441, and Asn445.

The structural annotation of the potentially candidate sugar transporter derived from sugar transportome is also an important step towards the understanding of their functions. The 3D-structural modeling, transport pathway, and network analysis were undertaken for this purpose. In this work, we selected the CCM_06358 as a representative member of pentose transporters for the structural modeling. At first, the crystal structure of Sugar Transport Protein 10 (STP10; PDB ID: 6H7D) [51] was chosen as a template for the 3D-modeling under sequence identity of 36%. As an essential progression of model building, original refinement of the loop conformation after model generation was automatically performed by Modeller Loop refinement-DOPE-Loop method during the process. The modeled 3D-structure of CCM_06358 was then selected on the grounds of the least DOPE score of −65,151.15. As shown in Figure S1, the psi and phi distribution of the Ramachandran plot for the modeled CCM_06358 shows that 92.8% of residues were present in the most favorable regions, and 5.3% of residues existed in the additionally allowed regions. There were 0.6% of the residues placed in generously allowed regions, and 1.3% of them were located in the disallowed region. As a result, the CCM_06358 was annotated as a putative monosaccharide transporter belonging to the major facilitator superfamily (TC: 2.A.1.1). Similar to other transporters, the modeled CCM_06358 was composed of 12 TM helices, TM1–TM12, connected by six extracellular loops and six intracellular loops (Figure 5A). It should be noted that individual TM7, TM8, and TM10 were broken into two segments, designated as ‘TM7a–TM7b’ and so on.

For probing the structure and function of sugar transporters on targeted carbon molecules, identification of tunnels is the next key step. In order to predict the theoretical position of xylose when binding with CCM_06358, Caver Web 1.0 [52] was used to predict a catalytic pocket located at the TM1 and TM2 (residues 38 to 69) with high druggability of 0.89 (Figure 5B). The xylose at this opening position was docked, and the binding pose showed that the xylose interacted with Gln50, Met311, and Thr315 through H-bonds (Figure S2A). The significant binding affinity of −19.54 kJ/mol indicated that the docking of xylose was reasonable. Furthermore, the stability of the complex was investigated by MD simulation with lipid membrane environment (Figure S3A). The RMSD values of CCM_06358 complex increased to 0.40 nm, which were relatively stable after 50 ns (Figure S3B). During the first 20 ns, the RMSD values were increased to 0.35 nm and stable. This simulation showed that the pore region of the conformation was well-behaved and rather stable after 50 ns.

Furthermore, the CAVER 3.0 tool [42] was used to detect transport pathway of CCM_06358 from a predefined starting point, by finding cheapest path towards the protein surface based on a cost function accounting for diameter and length. Alternatively, a Lid domain containing a conspicuous cluster of aromatic residues served as “doors” for substrate entry in the primary model of STP10 [41]. By aligning between the modeled and the template structures, the structurally conserved residue was chosen i.e., Gln50 as a starting point for the tunnel computation. By setting probe radius of 0.8 Å using similar size of pentose sugars, the result of CAVER analysis could predict 43 transport pathways in CCM_06358. Among these pathways, we noticed that the 25th, 27th, 37th, 39th, 41st, and 42nd pathways shared the same four bottleneck residues, including Phe38, Tyr85, Trp441, Asn445, which were of particular importance. However, the lengths of the 25th and 27th tunnels were too short and thus the remaining pathways (37th, 39th, 41st, and 42nd) were considered as candidate tunnels for CCM_06358. The 3D-visualization of the four candidate tunnels is shown in Figure 5B, and the detailed exploration of these tunnels, including location, throughput, cost, bottleneck radius length, curvature, as well as bottleneck residue are listed in Table 2. Among these four tunnels, they shared several residues including bottlenecks from the starting point, where they were separated near the broken helices region (Figure 5C). It is also interesting to investigate whether xylose can bind to the bottlenecks. Hereby, the docking result showed that xylose was able to form H-bonds with residues Tyr85 and Asn445 with binding energy of −26.65 kJ/mol (Figure S2B). In detail, the end of the 37th tunnel (the red pathway) through the space between the TM7a and TM8b, the 39th tunnel (the green pathway) ended through the space between the TM7a and TM11, the 41st tunnel (the pink pathway) ended through the space between the TM7a and TM10b, and the 42nd tunnel (the blue pathway) ended through the space between the TM2 and TM11. The spatial locations of four candidate tunnels highlighted the role of broken helices, which may help to control the size of the entrance at the starting point of the tunnel and also guide the direction when transporting the specific sugar through tunnels.

### 3.6. Network Analysis Identified Peculiar Residues of CCM_06358 with a Key Role in Transport Pathways

In order to further characterize the transporter tunnels, the RIN method was applied to predict key residues and interactions in the sugar transport pathway. RIN is a novel graph-based approach, which has assisted in unveiling the role of several kinds of importantly functional residues. In the framework of RIN, amino acid residues in the protein are referred to as nodes that are connected by edges over the non-covalent interactions [53,54]. The betweenness centrality of a node is defined as the number of the shortest path that passes through that node in the network, representing a global measure of the node contribution to the communication within the network. The betweenness centrality can be exploited for characterizing and differentiating the highly connected residues that contribute to stable interaction networks and allosteric communications in protein structures [55,56]. Herein, we employed the network parameters to predict network bottlenecks in CCM_06358 of *C. militaris*.

**Figure 6:**
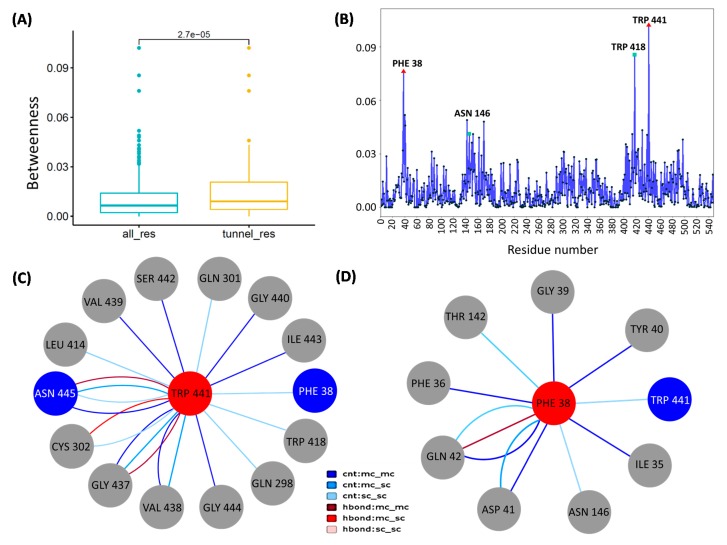
Analysis of residue interaction networks of CCM_06358. Note: (**A**) The distributions of betweenness for all residues and tunnel residues in CCM_06358. (**B**) The residue centrality profile of CCM_06358, while two tunnel bottlenecks with high betweenness are indicated by red triangles. (**C**) A subnetwok associated with the bottleneck residue of Trp441 in CCM_06358. (**D**) A subnetwok associated with the bottleneck residue of Phe38 in CCM_06358. Edges that represent contacts (cnt) are colored in blue; hydrogen bonds (hbond) are indicated in red, while darker and lighter colors stand for main chain (mc) and side chain (sc) atom interactions.

In Figure 6A, the box plot shows the difference between the residues placed in the four candidate tunnels and all residues, revealing that the tunnel residues had higher betweenness, where the mean values of betweenness of all residues and tunnel residues were 0.0097 and 0.014, respectively, under the *p*-value of 2.7E-05 by Wilcoxon signed-ranked test. The obtained betweenness values indicated a significant difference between two sets of residues, referring that the betweenness centrality might act as a potential in-silico marker for tunnel residues. Figure 6B shows the profile of betweenness centrality in CCM _06358. By mapping tunnel bottlenecks onto the centrality profile, observably Phe38 and Trp441 (red triangles) located at the peaks of the profile were found, while the Trp441 showed the largest betweenness centrality. In addition, two other tunnel residues, Asn146 and Trp418 (green squares), showed the other two peaks. Accordingly, the results of both tunnel and network analysis indicated that Phe38 and Trp441 might act as hotspots form bridges of transport pathways, since these two residues served as pathway and network bottlenecks.

Subsequently, the local network topology at bottleneck residues for predicting the local interactions was characterized. Not only the bottleneck residue in the pathway and network, but Trp441 also showed the second largest value of degrees, which suggests that Trp441 can interact with a large number of local residue neighborhoods. As a result, Figure 6C shows the local network of Trp441 which is associated with the other two bottleneck residues, Phe38 and Asn445. This network module containing three out of four bottlenecks might play important roles in the transport pathways. Among them, the two interactions (Phe38-Trp441, Trp441-Asn445) might create a pivotal function. Besides, Trp441 had multiple edges with Asn445 and Gly437 which displayed stronger and more impact interactions. Notably, Phe38 was another bottleneck residue with relatively high betweenness. In comparison with Trp441, Phe38 had no high degree which constituted a smaller local network (Figure 6D). Expectedly, as abovementioned, an important interaction between Phe38-Trp441, here Phe38-Asp41 and Phe38-Gln42 interactions, contain double and triple edges, respectively. Apart from the significance of single residue (Phe38) as a xylose recognition site [47], the amino acid interactions identified by molecular modeling might refer to the structure–function relationship between the protein transporter and the targeted sugar molecule. However, the exact role in such amino acid interactions is still undisclosed. Thus, we could simply raise a hypothesis that the two residues, Phe38 and Trp441, together with their important interactions would provide clues in the future study of site-directed mutagenesis to improve the xylose transport efficiency.

### 3.7. Verification of Functional Roles of Key Residues of CCM_06358 by Binding Free Energy Calculation

The MM-PBSA method was applied to calculate binding free energy and further evaluate the relative stability and molecular interaction of the xylose to CCM_06358. Firstly, the total binding energy and the separate energy component of xylose to CCM_06358 at the bottleneck position predicted by docking was calculated based on MD trajectories from 50 to 100 ns, which are listed in Table S4. It was observed that the calculated binding free energy of xylose to CCM_06358 was −19.60 ± 9.88 kJ/mol. The results indicated that xylose could form stable interactions with CCM_06358 at the bottleneck position. The energy of van der Waals mostly contributed to the xylose binding (ΔE_vdw_ = −60.97 ± 11.74 kJ/mol), which was in accordance with the interaction analysis because this complex showed a higher number of hydrophobic interactions.

In order to reveal the molecular interaction of xylose and CCM_06358, the CCM_06358 residues with possible interaction with xylose molecules were determined. The g_mmpbsa tool decomposes the total binding energy into the contribution made by each residue. Table 3 shows a list of the top 10 residues that contributed to the overall binding energy (ΔG_bind_). Observably, the hydrophobic residues with strong interaction with xylose were Ile35 and Phe38 in TM1, Ile174 and Val177 in TM5, and Trp418 in TM10, which were located around the bottleneck position. Mostly, no charged/polarized residues were found to interact with xylose, suggesting that a stability of the CCM_06358 and xylose complex was established due to hydrophobic interaction. Among them, three important bottleneck residues predicted by the high betweenness node in network analysis were also found, including Phe38, Trp418, and Trp441, where Trp418 and Phe38 had the largest free energy contributions. Accordingly, the binding free energy calculation results based on MD simulation could verify functional roles of such key residues of CCM_06358.

## 4. Discussion

Recent advances in the multi-level omics through integrative data analysis have opened the way for structure-based modeling and simulations, which would provide a mechanistic understanding of their transport function and interactions. In this study, the integrative analysis of sugar transportome and global transcriptome could be used to identify CCM_06358 as a potentially candidate sugar transporter. In addition to the upregulated expression pattern of CCM_06358 in the xylose culture, interestingly, the two conserved structural motifs responsible for xylose recognition were found at the transmembrane helices of CCM_06358, including Phe38-Gly39-Tyr40-Asp41-Gln42-Gly43 (FGYDQG) and Tyr312-Gly313-Pro314-Thr315-Ile316-Phe317 (YGPTIF). These finding suggest that the transport function of CCM_06358 might directly involve in xylose utilization in *C. militaris* [47]. Towards structural analysis, the homology modeling and molecular docking of CCM_06358 provided the 3D-structure of this transporter, whose stability could be investigated by MD simulation. Although the integration of structural and docking methods with omics data analysis have been proposed by Shen et al., 2019 [57], our computational pipeline first employed the tunnel and network analysis to the end of key residues and interactions using the data from sugar transportome, which could overcome the limitation in the common structural analysis. Therefore, the tunnel analysis could facilitate the identification of some potential transport pathways for xylose molecule, while the network analysis permitted the prediction of important bottleneck residues, including Phe38, Trp418, Trp441, and Asn445, and their interactions shared by different tunnels. At last, the binding free energy calculation was employed to verify the functional significance of three out of the predicted four residues (Phe38, Trp418, and Trp441). Taken together, a strategic flowchart of how to connect sugar transportome data with their structural role by 3D-modeling, and tunnel, network, and binding free energy analysis is thus proposed in this work as shown in Figure 1.

Our results of sugar transportome and global transcriptome analysis could suggest that CCM_06358 might be a putative transporter for xylose. The structural modeling, together with molecular docking and MD simulation results also showed that the complex structure of the binding of CCM_06358 and xylose was stable. The importance of key residues of CCM_06358 for transporting xylose was not only further verified by the docking and binding free energy analysis with significant affinities, but also some of whose functions have been reported in the previous experimental analysis. Our finding from the structural analysis coincides with the previous experimental study, documenting that Phe38 functions as a xylose recognition site [47,58]. It has been reported that the substitution of Val38 residue by Phe38 residue could be rewired the hexose transporter into xylose transporter leading to efficient xylose utilization in yeast [47]. In addition, the findings obtained from the integrated computational approach through the conserved motifs alignment and tunnels analysis in this work supports the functional roles of two conserved motifs of CCM_06358 in the xylose transport. The first motif of FGYDQG belonging to the tunnel bottleneck residues, and the second motif of YGPTIF together with our identified catalytic pockets were crucial for constituting the door of the tunnel of putative xylose transporter of *C. militaris*. Accordingly, the network and bottleneck analysis towards docking analysis could suggest some key residues and their interactions in the xylose transport function. Overall, it is apparent that our strategy of multi-level omics analysis and molecular modeling with transport pathway and residue interaction network analysis used here generated remarkably meaningful information in biological contexts of the xylose transporter as a case study of *C. militaris* that would be applicable to intensively study the sugar uptake process in the fungal platform at a system level. 

## 5. Conclusions

In this work, we present the first comprehensive study on sugar transportome of *C. militaris*, consisting of 85 sugar transporters enabling acquisition of diversified sugars for fungal growth. The transcriptional response of the identified sugar transporters on different carbon sources confirmed their genomic functions existed, and also reflected their biological relevance that permitted the identification of the candidate pentose transporters. One of them, CCM_06358, was elucidated to display a functional role in pentose transport of *C. militaris*, as a result of its 3D-structural organization attributing xylose recognition, in which the key amino acid residues (Phe38 and Trp441) were crucial in interactions with other residues. Additionally, this study provides a perspective in the context of “from sugar uptake to cordycepin production” in *C. militaris*, suggesting the presence of cellular mechanisms controlling the cordycepin production at multiple levels, not only the transcriptional control of metabolic genes as previously reported. The strategy used herein expanded remarkably valuable information, which is applicable for the study of sugar transportome in fungal systems and the structure engineering of targeted transporter proteins that might link to the production of bioactive compounds derived from xylose metabolism, such as cordycepin.

## Figures and Tables

**Table 1:** Characteristics and consensus features of sugar transportome in *C. militaris*.

Consensus Features	Numbers of Sugar Transporters
Protein domain (PFAM)	*49*
PF00083: Sugar transporter	49
Protein families (InterPro)	*65*
IPR003663: Sugar/inositol transporter	41
IPR004853: Sugar phosphate transporter	8
IPR005828: Sugar transporter	9
IPR005829: Sugar transporter	5
IPR007271: Nucleotide-sugar transporter	1
IPR011701: Major facilitator superfamily	1
Eukaryotic orthologous groups (KOG)	*63*
KOG0254: Predicted transporter	48
KOG1444: Nucleotide-sugar transporter	2
KOG1441: Carbohydrate transporter	6
KOG2234: UDP-galactose transporter	1
KOG0252: Inorganic phosphate transporter	3
KOG0769: Mitochondrial carrier protein	1
KOG4332: Sugar transporter	1
KOG0569: Carbohydrate transporter	1
Transporter classification database (TCDB)	*75*
2.A.1: Major facilitator superfamily	58
2.A.2: Glycoside symporter family	3
2.A.7: Drug/metabolite transporter superfamily	9
2.A.16: Dicarboxylate transporter family	1
2.A.29: Mitochondrial carrier superfamily	1
2.A.50: Glycerol uptake transporter family	1
2.A.96: Acetate uptake transporter family	2
Total of non-redundant sugar transporters	85

**Table 2:** Characteristics of the four candidate tunnels of CCM_6358 identified by CAVER 3.0 [42].

Tunnel	Location	Throughput ^1^	Cost	Bottleneck Radius ^2^	Length ^3^	Curvature ^4^	Bottleneck ^5^ Residue
37	Between the TM7a and TM8b	0.039658	3.227465	0.855433	84.94969	1.64852	Phe 38; Tyr85; Trp441; Asn445
39	Between the TM7a and TM11	0.029718	3.515996	0.855433	85.94388	1.555505	
41	Between the TM7a and TM10b	0.024121	3.724671	0.855433	94.43239	1.576965	
42	Between the TM2 and TM11	0.017327	4.055483	0.855433	98.90193	2.068183	

Note: ^1^ Probability that the pathway is used as a route for transport of the substances using the formula. ^2^ Maximal probe size which can fit in the narrowest part of the tunnel. ^3^ Length of the tunnel from the starting point to the protein surface. ^4^ Shape of the tunnel as the ratio between the length of the tunnel and the shortest possible distance between the starting point and the tunnel ending point. ^5^ The narrowest part of the tunnel (bottleneck) including a list of surrounding residues and a static picture of the bottleneck with the tunnel visualized as spheres and surrounding residues as sticks.

**Table 3:** Decomposition of binding free energy (kJ/mol) on per-residue basis of CCM_06358.

Residues	ΔE_MM_	ΔG_polar_	ΔG_SASA_	ΔG_bind_
Trp418	−4.377	2.3992	−0.4297	−2.4067
Phe38	−2.7748	1.1358	−0.3484	−1.9848
Ile174	−1.4312	0.2321	−0.2701	−1.4684
Ile35	−1.4563	0.3508	−0.2033	−1.3111
Val177	−0.9842	0.2892	−0.1434	−0.8382
Ile306	−1.2814	0.7336	−0.1411	−0.69
Trp441	−2.524	2.0924	−0.2414	−0.6728
Pro150	−0.81	0.3465	−0.0892	−0.5512
Leu414	−1.9582	1.6616	−0.1454	−0.4428
Ala307	−0.7957	0.5096	−0.1578	−0.442

Note: ΔE_MM_, ΔG_polar_, and ΔG_SASA_ are binding energy components of energy in vacuum, polar, and nonpolar solvation energies, respectively. ΔG_bind_ is the total binding energy.

## Supplementary Materials

The following are available online at https://www.mdpi.com/2073-4409/9/2/401/s1. Table S1: A list of known/putative sugar transporter sequences from different fungi which was used for structural motif analysis.

Table S2: A list of consensus features of sugar transportome of *C. militaris*. Table S3: A list of significantly expressed sugar transporter genes across three experimental sets of the *C. militaris* sucrose, glucose, and xylose cultures. Table S4: The total binding energy and its components of CCM_06358 as well as xylose complex obtained from g_mmpbsa tool. Figure S1: Ramachandaran plot of CCM_06358. Note: There was more than 98% of residues in the allowed region suggesting the high quality of the structure. Figure S2: Molecular docking results for xylose binding with CCM_O6358. Note: (**A**) The bonding pose of xylose at the entrance position, consisting of Gln50, Met311, and Thr315; (**B**) the bonding pose of xylose at the bottleneck position, consisting of Tyr85 and Asn445. Figure S3: Molecular dynamics (MD) setup and RMSD results for simulating the interaction of CCM_06358 and xylose. Note: (**A**) A representative CCM_06358 conformation observed in simulation. The CCM_06358 structure (orange) constructed by using homology which was embedded into a POPE lipid bilayer (green). The substrate xylose (purple) was initially placed on the bottleneck sites indicated by caver. Water molecules were displayed in red lines; (**B**) the RMSD of all Cα atoms of CCM_06358 throughout 100 ns MD simulation for the complex.
