# Supplementary figures and images for: Probing Carbon Utilization of Cordyceps militaris by Sugar Transportome and Protein Structural Analysis
Source: Cells. 2020 Feb 10;9(2):401. doi: 10.3390/cells9020401 (PMC7072658; doi:10.3390/cells9020401)

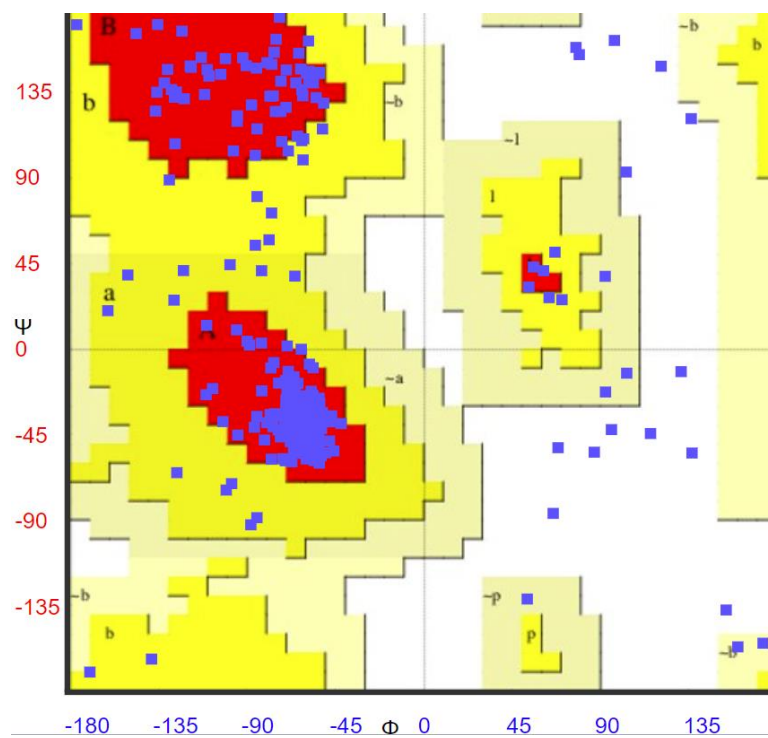

Figure S1.

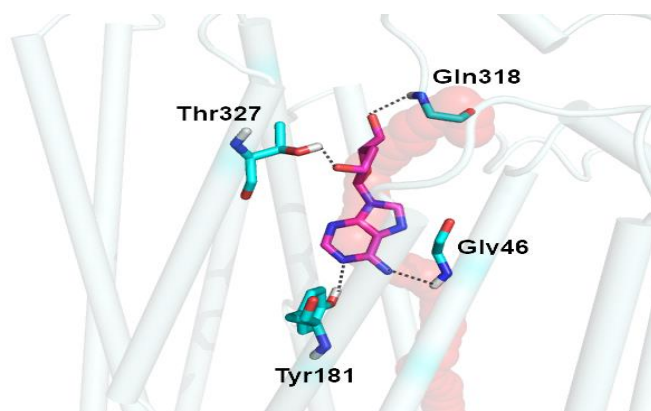

(A)

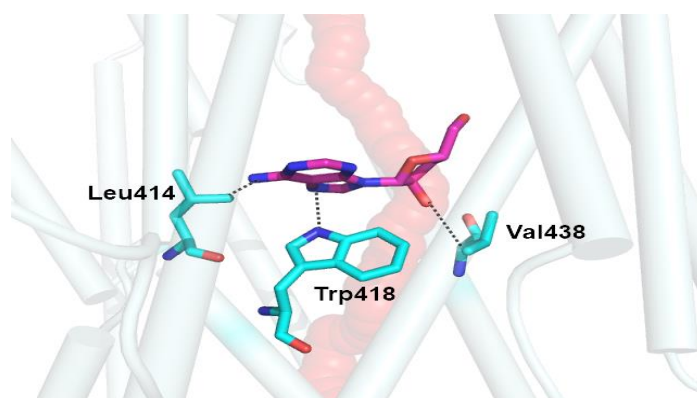

(B)

Figure S2

Supplement: Supplementary file 1 [file cells-09-00401-s001.zip › cells-673666-supple-final1.pdf]
